# Only minimal regions of tomato yellow leaf curl virus (TYLCV) are required for replication, expression and movement

**DOI:** 10.1007/s00705-014-2066-7

**Published:** 2014-04-10

**Authors:** Ofer Gover, Yuval Peretz, Rita Mozes-Koch, Eyal Maori, Haim D. Rabinowitch, Ilan Sela

**Affiliations:** The Robert H. Smith Faculty of Agriculture, Food and Environment, The Hebrew University of Jerusalem, 76100 Rehovot, Israel

## Abstract

The IL-60 platform, consisting of a disarmed form of tomato yellow leaf curl virus (TYLCV) and auxiliary components, was previously developed as a nontransgenic universal vector system for gene expression and silencing that can express an entire operon in plants. IL-60 does not allow rolling-circle replication; hence, production of viral single-stranded (ss) DNA progeny is prevented. We used this double-stranded (ds) DNA-restricted platform (uncoupled from the dsDNA→ssDNA replication phase of progeny viral DNA) for functional genomics studies of TYLCV. We report that the noncoding 314-bp intergenic region (IR) is the only viral element required for viral dsDNA replication. None of the viral genes are required, suggesting recruitment of host factors that recognize the IR. We further show that IR-carrying reporter genes are also capable of replication but remain confined to the cells into which they were introduced. Only two sense-oriented viral genes (*V1* and *V2*) need to be added to the IR-carrying construct for expression and movement. Hence, any IR-dsDNA construct supplemented with *V1* and *V2* becomes a replication-competent, mobile and expressing plant plasmid. All viral functions (replication, expression and movement) are determined by the IR and the sense-oriented genes. The complementary-oriented viral genes have auxiliary roles in the late phase of the virus “life cycle”. The previously reported involvement of some viral genes in expression and movement is therefore revised.

**Electronic supplementary material:**

The online version of this article (doi:10.1007/s00705-014-2066-7) contains supplementary material, which is available to authorized users.

## Introduction

Tomato yellow leaf curl virus (TYLCV) is a monopartite begomovirus with a characterized genome organization consisting of six overlapping transcribed open reading frames (ORFs) [1–4]. Briefly, TYLCV consists of six overlapping open reading frames (ORFs) transcribed in opposite directions from two promoters situated at either end of an intergenic region (IR). The 314-bp IR carries the universal motif TAATATT/AC. In addition to carrying promoters, it also serves as the viral origin of replication. It carries motifs (iterons) for binding the replicase-associated protein (REP—the product of ORF *C1* [1]). Two ORFs are expressed in viral (sense) orientation—*V2* and *V1* [pre-coat and coat protein (CP), respectively]—and four ORFs in the complementary orientation (*C1* to *C4*).

Geminiviral DNA replication involves a number of steps [2, 6]. The uncoated open circular single-stranded (ss) DNA converts into a covalently closed circular double-stranded (ds) DNA. In begomoviruses, this stage requires priming by RNA, with the primosome complex provided by the host [3, 6]. The conversion of covalently closed circular DNA into supercoiled forms is carried out solely by the host enzymatic machinery [5, 7, 8]. Closed circular dsDNA forms are bound by host histones to form minichromosomes, leaving sufficient gaps at the origin of replication, the promoter sites and the iterons to allow interactions with plant proteins and REP [1]. The dsDNA within the minichromosomes replicates and is used for transcription of the viral genes. When a sufficient amount of REP becomes available, it binds to the IR iterons, initiates a specific nick for rolling-circle replication, and produces the ssDNA-molecule progeny.

The viral genes *V1*, *V2*, *C4* [2, 3, 5, 6] and (indirectly) *C2* [1] are involved in symptom development and expression, disease severity and movement [2, 3, 5–7]. Trafficking of the geminiviral genome within a cell, between cells and over long distances within the host has been attributed in some cases to the viral ssDNA [1–3, 6]. In other cases, both ssDNA and dsDNA have been implicated as moving entities in a non-sequence-specific manner [9].

The CP carries nuclear-localization and nuclear-export signals [11–16] and is thus involved in facilitating ssDNA invasion of the nucleus and possibly directing viral nuclear components back to the cytosol. *C3* and *C*2 code for factors involved in replication and transcription enhancement, respectively.

Thus, five of the viral ORFs have no direct role (but some auxiliary roles) in viral dsDNA replication. The sixth ORF (*C1*) is only essential for rolling-circle replication, a late-phase function which may, in turn, provide a template for dsDNA replication and play some auxiliary roles in recruiting plant proteins for optimization of viral replication and expression.

In a previous study, the IL-60 platform of TYLCV-derived plasmids (see further on for construct definitions) was found to be capable of expressing foreign genes or silencing native plant genes in a nontransgenic manner in every plant tested [17]. Moreover, it can harbor long inserts and express an entire operon [18]. In the IL-60 system, the activity of the ORF *C1* (REP) product is compromised, thus eliminating rolling-circle replication and the production of progeny ssDNA. Therefore, the inserted IL-60 DNA components replicate in plant cells only as dsDNA.

In our previous study [17], we further demonstrated that a foreign gene could be placed within the IL-60–pBlueScript (BS) construct or under IR control on a different construct for expression. In the latter case, IL-60–BS served as the “driver”, providing needed elements for replication and expression [17]. In the present report, we show that p1470, carrying the IR and only two sense-oriented TYLCV genes (without any of the four complementary-oriented genes), can replace the larger construct, IL-60–BS, in driving expression of a foreign gene in *cis* as well as in *trans*.

Here, the IL-60 system (including its various components) was used to study aspects of TYLCV functional genomics. A major discovery was that the short noncoding viral DNA segment (IR) is sufficient for basal levels of viral replication and that the addition of only two viral genes (IR–*V2–V1* = p1470) allows expression and movement. It was also found that a reporter gene is expressed in *cis* and in *trans* when fused to p1470 or placed under IR control with p1470 serving as the driver (the limited-size construct p1470 could replace IL-60–BS as a driving entity in transactivation of IR-X). This paper demonstrates that the IR is fundamental to viral replication, expression and movement and, at a later phase, is regulated to function (along with other viral and host factors) in enhancing progeny production. The association of TYLCV CP with IR is also demonstrated.

## Materials and methods

### Clones and constructs

In this report, shorter components of the previously reported IL-60 system [17] were explored, as described in the section “IR-carrying constructs” below. The term “IL-60 system” is used when we are not referring to a particular component of the system. “IL-60–BS” refers to a manipulated construct of TYLCV fused to the plasmid BlueScript, 5,682 bp in length, as described by Peretz et al. [17]. All constructs not carrying IL-60-BS were built for the resent study or their use was largely elaborated in the present paper. In some of the constructs described here and in our previous report [17], the BS plasmid could be replaced by another plasmid (pDRIVE).

**Fig. 1:**
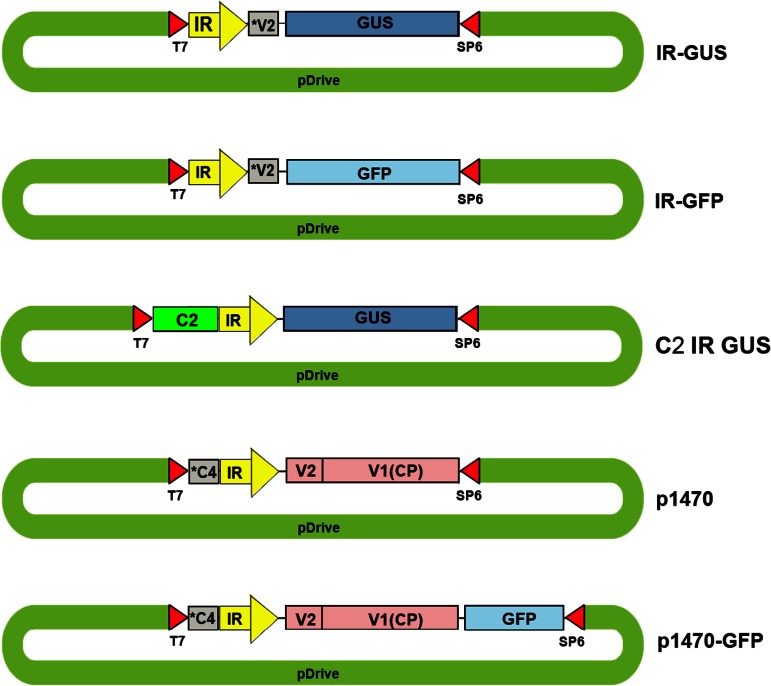
Illustrations (not to scale) of the various IR-carrying constructs. pT7 and SP6, sites of the T7 and SP6 promoters; *V2, the first 282 bp of ORF V2; C4*, the last 279 bp of the interrupted C1 ORF (the truncated N terminus of C4); C2, the entire ORF of TYLCV C2

The IR segment of TYLCV was PCR-amplified and cloned into the T/A cloning vector pDRIVE. This construct presumably carries the ribosomal binding site of *V2*. A β-glucuronidase (GUS) gene (*uidA*) was force-cloned with *Sal*I and *Sac*I into pDRIVE–IR downstream of the IR. Similarly, the gene for green fluorescent protein (GFP) was force-cloned with *Sal*I and *Hind*III. *C2* was cloned on the opposite side of GUS using *Pst*I and *Kpn*I (Fig. 1). In all constructs, IRs were positioned so that the transcripts would be regulated by the promoter controlling *V2–V1* transcription. 35S:GFP served as an IR-less control.

### Propagation of IL-60 constructs and their administration into plants

*Escherichia coli* cells were transformed with the pertinent IL-60 construct and propagated under ampicillin selection, and the construct was extracted by standard procedures [19]. IL-60 constructs were administered to plants by injection [17] or root uptake—root tips of tomato plantlets at the first leaf stage were trimmed and immersed for 1–2 days at room temperature in an aqueous solution containing 1 µg DNA per plant until solution imbibition was complete.

### TYLCV inoculation

TYLCV-viruliferous *Bemisia tabaci* was maintained on TYLCV-infected *Datura stramonium* or tomato plants. For inoculation, viruliferous insects (50 insects per plant) were placed in cages containing uninfected, IL-60-carrying tomato plants at the second-true-leaf stage. A week later, the insects were killed using Confidor and the plants were transferred to an insect-free cage. A week after that, all plants were inspected for residual insect infestation, and all insect-free plants were transferred to the greenhouse for phenotypic observation and molecular analysis.

### Molecular analysis

Southern, northern, western, PCR, quantitative (q) PCR and RT-PCR analyses were carried out according to standard procedures [19 and manufacturers’ protocols]. Unless stated otherwise, PCR assays consisted of 40 cycles. Probes for Southern and northern analyses were labeled by the PCR-DIG procedure (Roche Molecular Biochemicals). GUS expression was monitored by staining according to Jefferson et al. [20]. Western blot analysis of plant material was performed on total tomato protein extracts after removal of the masking Rubisco protein according to Xi et al. [21].

Total DNA from BY-2 protoplasts was subjected to overnight digestion by *Dpn*I (Fermentas) in the original buffer at 20 units per 5 µl of genomic DNA. Digested DNA was PCR-amplified using primers for the entire length of the GUS gene (*uidA*) (ESM Table 1).

### Protoplast extraction and transformation

BY-2 cells were grown in Linsmaier and Skoog medium in which KH_2_PO_4_ and thiamine–HCl were increased to 370 and 1 mg/l, respectively, and sucrose and 2,4-D were supplemented to 3 % and 0.2 mg/l, respectively. Cells were harvested 3 days after culture dilution to reach peak mitotic stage [22]. BY-2 cells were centrifuged at 500 rpm and the supernatant was gently removed. Cell walls were then digested in an enzymatic solution (1 % cellulose, 0.02 % pectolyase, 0.05 % bovine serum albumin, 0.025 M polyvinyl pyrrolidone [PVP] in 0.4 M sorbitol, 20 mM KCl, 20 mM 2-(*N*-morpholino)ethanesulfonic acid [MES], 10 mM CaCl_2_, pH 5.7) for 1.5 h at 30 °C. Debris was filtered out (Falcon cell strainer, cat. no. 352350), and the remaining cells were rinsed three times in isotonic solution. Extracted protoplasts, 10^5^ cell/ml, were suspended in MMg solution (0.4 M sorbitol, 15 mM MgCl_2_, 4 mM MES), incubated on ice for 30 min, and subjected to 25-min transformation with the relevant plasmid at 10 μg/100 μl in polyethylene glycol (PEG) solution (40 % PEG 4000, 0.4 M sorbitol, 0.1 M Ca(NO_3_)_2_) at room temperature. Following incubation, protoplasts were washed twice in W5 buffer (0.4 M sorbitol, 154 mM NaCl, 125 mM CaCl_2_, 5 mM KCl, 2 mM MES, pH 5.7), placed back in the growth medium and gently shaken in the dark for up to 72 h until microscopic observation and real-time PCR analysis.

### DNA extraction from BY-2 protoplasts

At each sampling, cells were removed and frozen at −80 °C, then placed in liquid nitrogen and homogenized in lysis buffer (100 µg/ml proteinase K, 0.4 % SDS) at 55 °C for 1 h using a mortar and pestle. DNA was extracted from the cell suspension with phenol:choloroform:isoamyl alcohol (25:24:1, v/v). Following centrifugation, the upper phase was decanted into a clean tube and supplemented with 1 volume of isopropanol and 20 µl 5 M NaCl. After another centrifugation the pellet was washed twice with 70 % EtOH and suspended in molecular-grade water.

### Confocal microscopy

Post-transfection, protoplast suspensions were studied over time under a confocal microscope (Zeiss 100M). Excitation was at 488 nm, and GFP emission was detected at 505–550 nm. Chlorophyll autofluorescence was detected at wavelengths longer than 560 nm. Data were processed by the built-in program LSM 51.

### Immunocapture PCR

Immunocapture PCR was carried out, with modifications, as described by Wetzel et al. [23]. Plant extracts (1 g leaves in 3 ml extraction buffer [20 mM Tris-HCl, pH 8.0, 138 mM NaCl, 3 mM KCl, 1 mM PVP, 0.05 % Tween 20]) were treated with DNase. Polystyrene microtiter plates or polypropylene tubes were coated with 100 μl anti-TYLCV antibodies (diluted 1:500 in carbonate buffer, pH 9.6) and incubated overnight at 4 °C. The wells were washed five times with PBS-Tween. Then, 100 μl of DNase-treated plant extract (diluted 1:100 in extraction buffer) was added and subjected to UV irradiation (12,000 J/cm^2^) for 4 h. PCR amplification was then carried out in the same wells with primers for TYLCV CP or phytoene desaturase (PDS) genes.

### Real-time qPCR

Each DNA sample was diluted 1:50 before amplification. Approximately 10 ng of DNA was taken for each PCR amplification. Real-time qPCR in a LightCycler 480 (Roche) was performed with the primers and probes listed in ESM Table 1, and data analysis was performed using the built-in software. The real-time PCR program was as follows: 95 °C for 10 min, followed by 45 cycles of 95 °C for 10 s and 60 °C for 30 s, ending with a step at 40 °C for 30 s. DNA levels were standardized using the 18S rRNA gene as an internal control. Plasmid DNA harboring the GUS gene was quantified using the standard calibration curve of serial 10-fold dilutions of IR–GUS plasmid. A tobacco 18S quantification curve was obtained using serial 10-fold dilutions of *Nicotiana tabacum* genomic DNA. Light Cycler software was used for actual quantification of each DNA species, and tomato actin was used as a standard. Primers and probes for GUS amplification and 18S DNA amplification are presented in ESM Table 1.

## Results

### IR-carrying constructs

For foreign gene expression, we cloned the reporter genes encoding GUS (bases 1466 to 3274, GenBank accession no. M14641) or GFP (excised from the plasmid 30b-GFP3, courtesy of William O. Dawson, University of Florida, Lake Alfred) downstream of the IR (IR-GUS and IR-GFP). IR–GUS was described previously [17] and is presented here by another illustration. IR–GFP is a similar construct, in which GFP replaces GUS. To assay the functions of the sense-oriented genes, we fused *V2–V1* to the IR, forming a TYLCV-derived 1,467-bp insert in pDRIVE, and named it “p1470”. Another construct named “p1470–GFP” consisted of the entire gene encoding GFP downstream of p1470. The bacterial plasmid component for all IR-carrying constructs was pDRIVE, but other plasmids, such as pBS, have also been employed successfully. In some cases, a nopalin terminator (termed *nos* or *ter*) was added to the reporter gene. In most cases, the tested genes were placed downstream to the “right” promoter of the IR (driving the expression of sense-oriented genes). In a certain limited number of constructs, genes were placed under the control of the “left” promoter of the IR. For example, in IR-C2-GUS, the C2 and GUS genes were placed under the “right” promoter, and in C2-IR-GUS, C2 was placed under the “left” promoter and GUS was placed under the “right” promoter. The constructs are diagrammatically represented in Figure 1.

### Foreign DNA longevity in plant cells is extended by the IR

We previously demonstrated foreign gene expression by plasmids consisting of a reporter gene downstream of the TYLCV IR when co-inoculated into plants with IL-60 [17]. Here, we tested the effect of the IR alone on the longevity (stability) of foreign DNA *in situ* in the absence of any detectable viral gene. To ensure ribosome binding to the RNA transcribed from the IR, the 5’ terminal sequence of *V2* (presumably carrying a ribosomal binding site) was fused between the IR and the reporter gene.

IR–GFP (Fig. 1) was injected into the petioles of 4- to 5-week-old tomato plants. In parallel, a GFP-carrying plasmid (pDRIVE) lacking the IR, as well as pDRIVE harboring GFP-coding sequences downstream of the 35S promoter, were simultaneously and similarly injected into control tomato plants. DNA was then extracted from the tissue excised from the treated site 1, 5, 10 and 20 days post-injection.

**Fig. 2:**
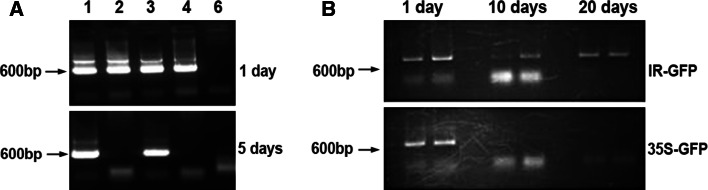
Stability of IR-carrying constructs in intact plants. All DNA samples were taken from the point of injection and were analyzed by PCR for the presence of GFP sequences. **A.** Stability of a GFP-harboring plasmid (pDRIVE–GFP) versus the same plasmid carrying the IR. Lanes 1 and 3, IR-carrying plasmids; lanes 2 and 4, IR-lacking plasmids; lane 6, negative control—DNA extract from untreated plants. **B.** Stability of 35S- vs. IR-carrying plasmids. PCR was performed at various times postinjection as specified

The constructs were administered into only a few cells, and therefore the presence of GFP sequences in the tissue samples could only be determined by PCR. Figure 2 shows that GFP sequences from all three constructs were present *in planta* for 1 day but only persisted for 5 days or more only in plants treated with IR-carrying constructs. We concluded that DNA stability is conferred by the IR but not by 35S, which is a strong virus-derived promoter for expression in plants.

### The IR is the only viral element required for basal dsDNA replication

**Fig. 3:**
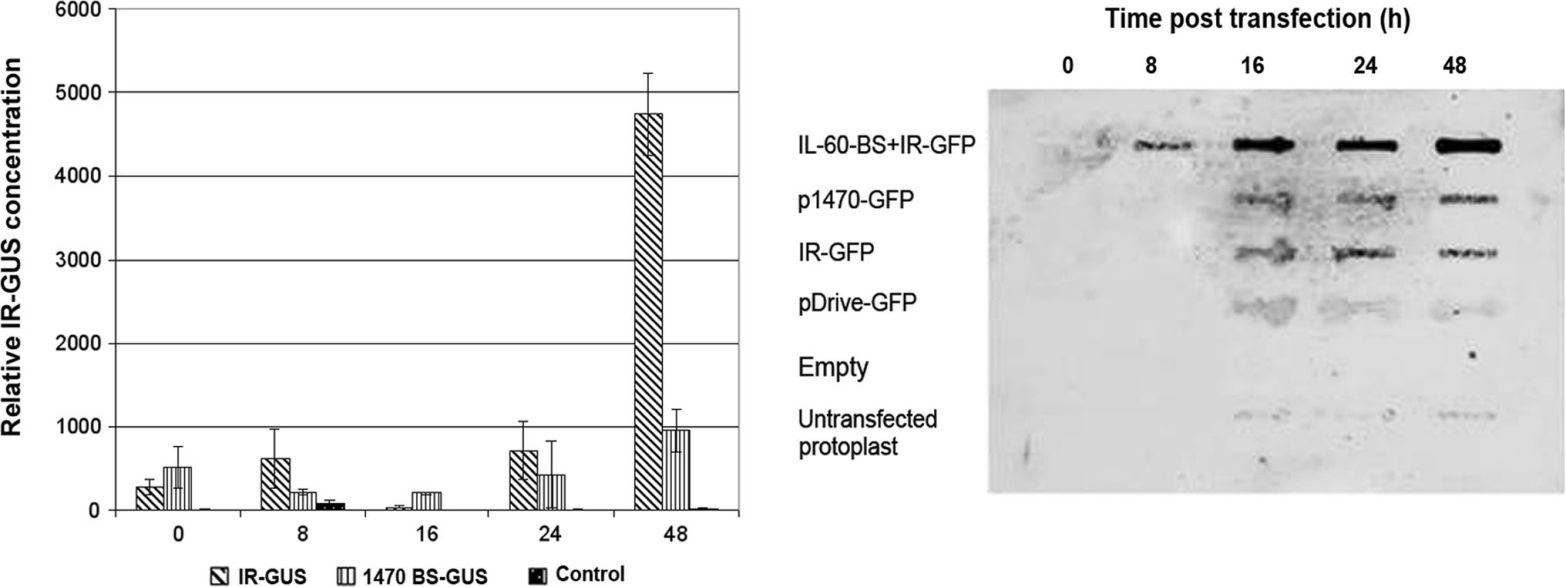
Left frame: Replication of IR–GUS in protoplasts as indicated by real-time qPCR. The time after application of the construct is shown on the x-axis. Real-time qPCR (average of three repetitions) was performed on DNA extracted from 10^5^ protoplasts, and results were normalized to those for the 18S rRNA gene. DNA extracted from untransfected protoplasts served as a control for real-time PCR. Right frame: Slot-blot assay of IR–GFP. The slot-blot was carried out by applying 10^5^ lysed protoplasts to each slot and hybridizing to a GFP probe

DNA constructs fused to the IR are stabilized in plant cells. This may result from, among other possibilities discussed further on, the ability of the construct to replicate in the cells. To test the potential for IR-directed replication, protoplasts were transfected with IR–GFP or IR–GUS. Slot-blot hybridization assay showed IR–GFP accumulation, indicating replication. This was corroborated by real-time qPCR, which showed replication of IR–GUS (Fig. 3). pDrive-GFP and 1470-BS-GUS served as IR-less controls. These results clearly show that GUS and GFP constructs carrying only the IR as the viral element accumulate with time in protoplasts. The level of DNA at zero time is considered to be the level of input DNA. Since the sensitivity of PCR is greater than that of hybridization, the level of input DNA is detected by PCR, while in the hybridization assay it is below the detection limit.

**Fig. 4:**
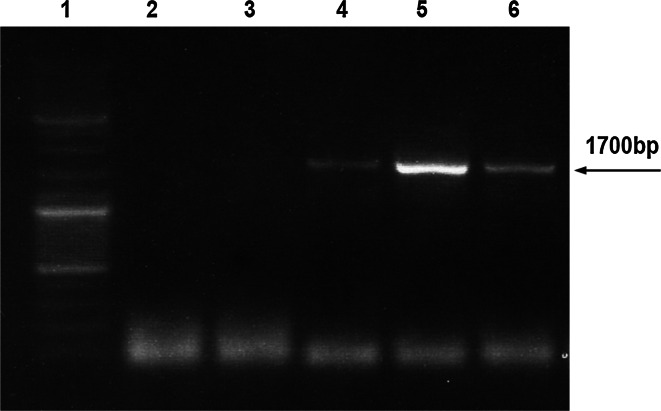
Evidence that the IR-GUS extracted from protoplasts is not methylated, indicating that it was replicated in the protoplasts and does not represent the input DNA. DNA extracted from protoplasts at various times following IR–GUS transfection was digested with *Dpn*I, and the entire *GUS* sequence was PCR-amplified. Failure of *Dpn*I digestion indicated that the DNA was not methylated. Lane 1, size markers; lane 2, control—DNA extracts from untransfected protoplasts; lane 3, digestion of bacterial-extracted IR–GUS; lanes 4–6, digestion of PCR-amplified DNA extracts from IR–GUS-transfected protoplasts at 16, 24 and 48 h posttransfection, respectively

Geminiviral DNA evades methylation in plant cells [24, and references within]. Therefore any geminiviral DNA made in plants is *Dpn*I resistant. Therefore, non-methylated geminiviral DNA indicates that it has been propagated *in planta* and not in bacteria. DNA replicated in protoplasts was resistant to *Dpn*I, indicating its plant (and not bacterial) origin (Fig. 4).

### Viral sense-oriented genes support IR-directed expression

**Fig. 5:**
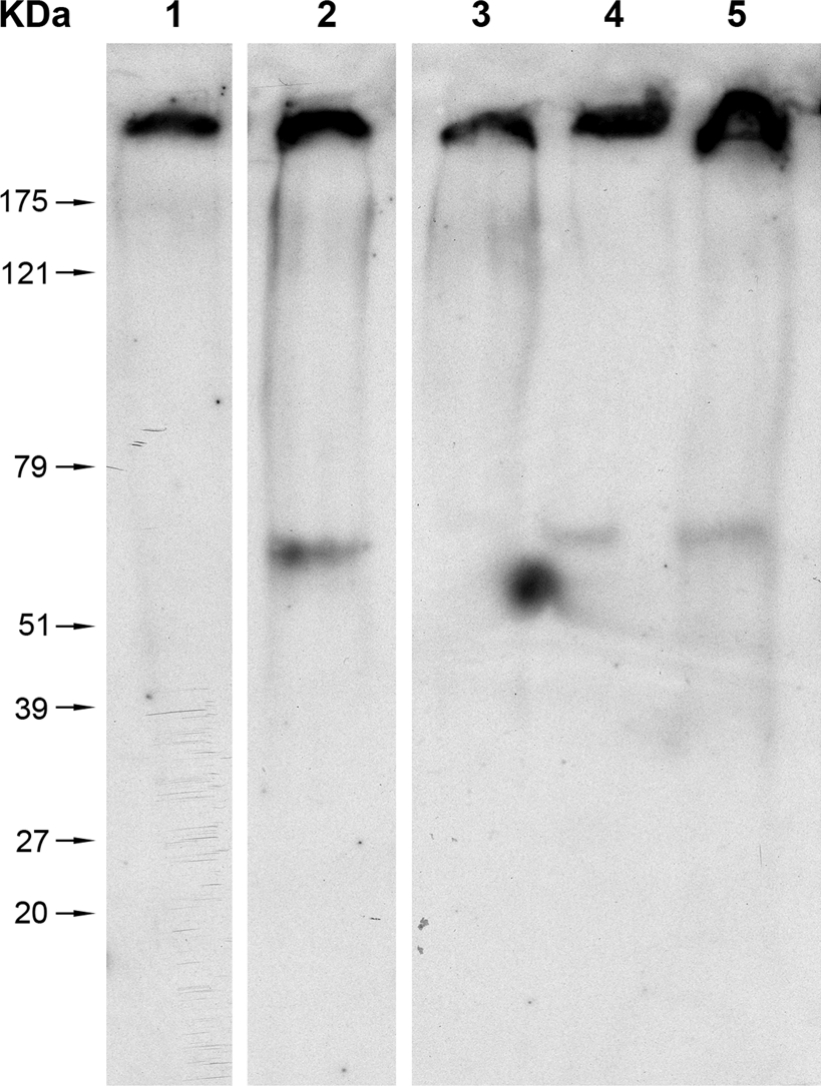
Western blot analysis for GUS. Lane 1, extracts of untreated plants; lane 2, extracts of plants treated with IL60+C2–IR–GUS–ter; lane 3: extracts of plants treated with IL60+35S:GUS–ter (no IR); lane 4, extract of plants treated with IL60+IR–GUS–ter; lane 5, extract of transgenic GUS-expressing plants

GUS protein was previously shown to be expressed from IR–GUS driven by IL-60 [17]. The western blot shown in Figure 5 illustrates that IR is necessary for GUS expression and cannot be replaced by the 35S promoter. The constructs were introduced into the plants by root uptake; proteins were extracted from plant leaves 2 weeks later, and the Rubisco protein was removed by polyethylene fractionation as described by Xi et al. [21]. The proteins were subjected to western blot analysis with anti-GUS antibodies (Fig. 5).

**Fig. 6:**
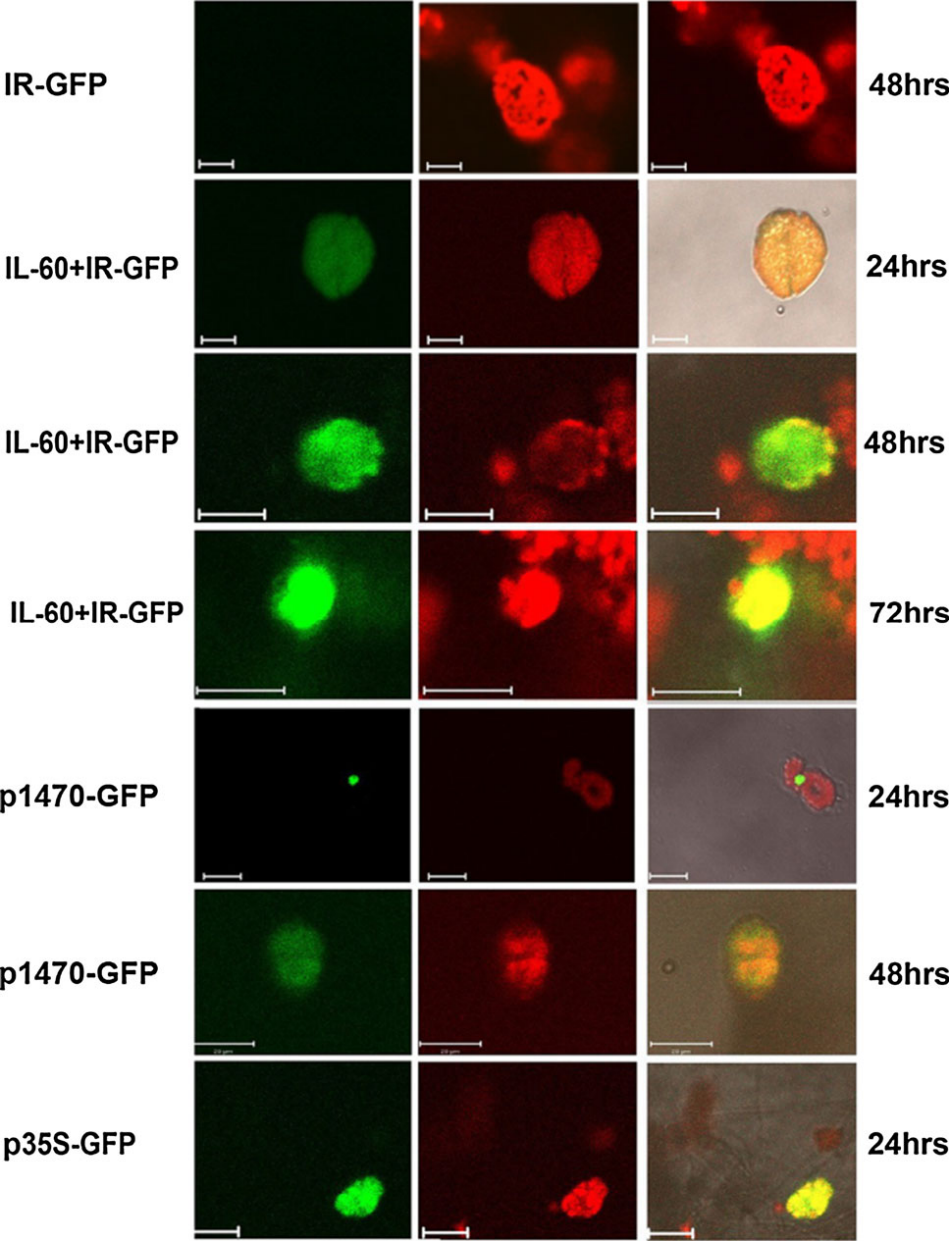
GFP expression in protoplasts transfected with various IL-60 constructs. GFP (left-hand column) and chlorophyll (center column) fluorescence was detected using a confocal microscope supplemented with suitable filters. Superpositions of the left-hand column on the center column are presented in the right-hand column

Expression was also followed *in vivo* by GFP fluorescence. IR–GFP alone, IR–GFP together with IL-60, and the fused construct p1470–GFP (carrying only sense-oriented TYLCV genes, as depicted in Figure 1) were introduced into protoplasts by transfection. IR alone did not support expression of the IR-fused reporter gene (Fig. 6). However, IR-directed expression did occur when IL-60 was added *in trans* or when the reporter gene was fused to the sense-oriented viral genes (*V2, V1*; exemplified by p1470–GFP). Adding p1470 to IR–GFP *in trans* also promoted expression (data not shown).

### TYLCV provides in-trans factors that facilitate mobilization of genes fused to the IR in plants

In our previous paper [17], TYLCV infection was shown to provide factors enabling the mobilization of IL-60–BS. Here, we demonstrate that the target of said factors is IR. GUS or GFP under the transcriptional control of the IR was injected into tomato leaf petioles. PCR amplification did not reveal any traces of the inserts in leaves remote from the point of injection.

**Fig. 7:**
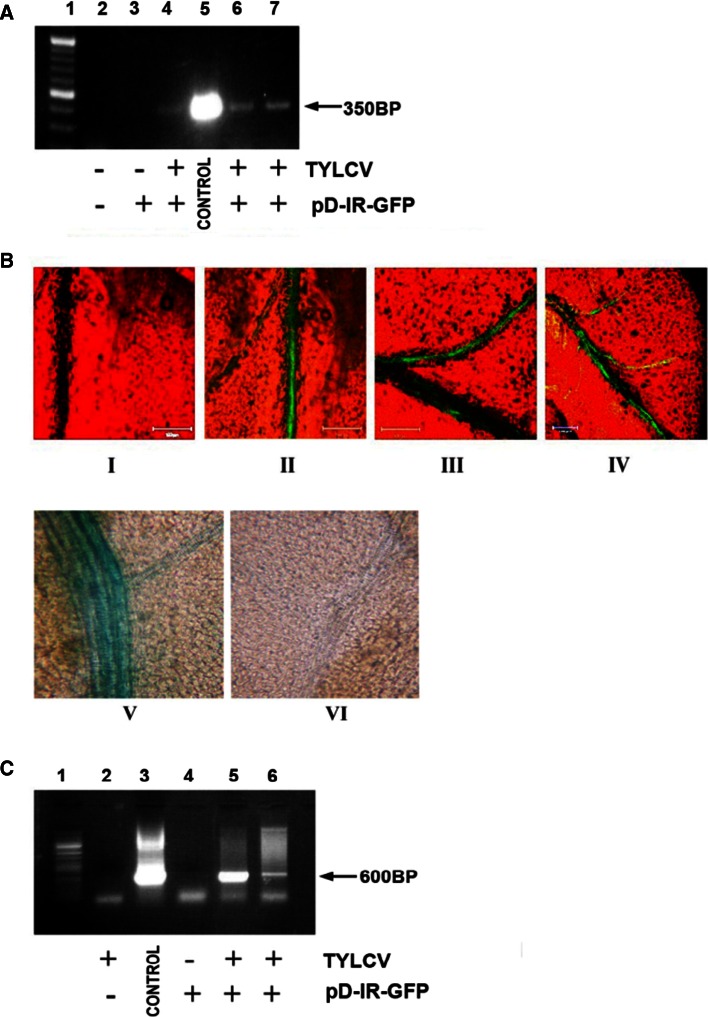
The effect of TYLCV infection on the expression and spread of IR-GFP. **A.** Detection of TYLCV in infected plant tissues by PCR, using TYLCV–CP primers. Lane 1, size markers; lane 2, DNA extract from untreated, uninoculated plants as template; lane 3, DNA extract from IR–GFP-treated, uninoculated plants as template; lanes 4, 6 and 7, DNA extracts from IR–GFP-treated, TYLCV-inoculated plants as template; lane 5, positive control—a plasmid carrying TYLCV CP served as the template. **B.** Expression and movement of IR–GFP and IR–GUS in TYLCV-infected plants. Confocal images were taken from leaves of IR–GFP-treated plants. I, TYLCV–inoculated, plasmid-devoid plant; II, plant treated with IR–GFP, 14 days after TYLCV inoculation; III and IV, plants treated with IR–GFP, 28 days after TYLCV inoculation. Bars represent 100 µm. V, image of a stained leaf section of an IR–GUS-treated plant, 14 days after TYLCV inoculation. VI, image of a stained leaf section of an untreated plant. **C.** Detection of GFP sequences (by PCR with GFP primers) in systemic leaves of IR–GFP-treated plants 28 days post-TYLCV inoculation. Lane 1, size markers; lane 2, DNA extract from a plasmid-devoid, TYLCV-infected plant as template; lane 3, positive control—a GFP-harboring plasmid served as a template; lane 4, DNA extract from IR–GFP-treated plant not inoculated with TYLCV as template; lanes 5 and 6, DNA extracts from IR–GFP-treated, TYLCV-inoculated plants as template; pD–IR–GFP, IR–GFP inserted in the plasmid pDRIVE

Seven days post-administration, the IR–GUS- and IR–GFP-treated plants were inoculated with TYLCV. Successful infection was confirmed by PCR for TYLCV CP (Fig. 7A). GFP distribution and expression in IR–GFP-treated plants were observed by fluorescence and PCR, respectively (Fig. 7BI–IV and 7C). Mobilization of IR–GUS in TYLCV-infected plants was detected by staining (Fig. 7BV). Note that addition of the sense-oriented *V1* and *V2* genes (in the form of p1470–X) supported mobilization *in planta*, which was comparable to what was observed in tissues infected with TYLCV (as reported further on).

### The IR and products of the sense-oriented viral genes *V1* and *V2* are sufficient for dsDNA mobilization in plants

IR-carrying constructs are capable of replication, but, as already noted, the products remain localized within the treated cells. The presence of TYLCV-mobilizing factors, however, enables *in vivo* movement through the treated plants. We defined *V1* and/or *V2* as the movement determinants by showing that, in addition to their role in directing expression, these sense-transcribed TYLCV genes under IR regulation can promote dsDNA mobilization in TYLCV-free plants.

**Fig. 8:**
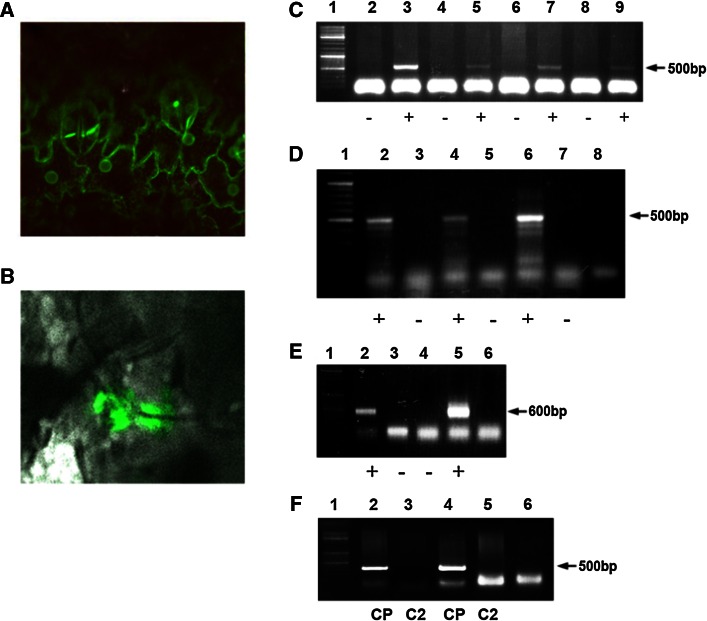
Replication and movement of p1470–GFP in tomato plants. **A.** GFP detection in systemic leaves of a plant treated with p1470–GFP. **B.** GFP expression in parallel locations across the cell wall in two adjacent cells. **C.** PCR with primers for TYLCV CP confirms the spread of p1470. (+) and (−) indicate p1470–GFP-treated and untreated plants, respectively. **D.** RT-PCR with primers for TYLCV CP, confirming the expression of *V1* (CP) from p1470. (+) and (−) indicate p1470–GFP-treated and untreated plants, respectively. Lane 8, negative control—PCR performed without template. **E.** PCR with GFP primers, corroborating the presence of p1470–GFP. (+) and (−) indicate p1470–GFP-treated and untreated plants, respectively. Lane 6, negative control—no template. **F.** PCR with primers for TYLCV C2. Two p1470–GFP-treated plants were analyzed for the presence of CP and *C2*. Lane 6, negative control—no template. The absence of *C2* indicates a lack of TYLCV contamination. Lane 1 in frames C–F, size markers

P1470–GFP was injected into the petiole of the second true leaf of tomato plants with 4–6 leaves. Four days later, the treated leaves and the younger leaves positioned above them were analyzed by confocal microscopy and PCR (Fig. 8). GFP fluorescence was evident in the cytoplasm of cells remote from the point of administration (Fig. 8A). GFP fluorescence was also observed in parallel locations in neighboring cells on both sides of the cell wall, suggesting cell-to-cell movement (Fig. 8B). In newly emerged leaves, expression and transport of GFP in the presence of *V2* and *V1* under IR control were confirmed by PCR and RT-PCR analyses (Fig. 8C–E). Figure 8F demonstrates the absence of TYLCV contamination, as indicated by the lack of *C2*. We propose that expression of *V1* and *V2* facilitated the mobilization of IR–GFP and its trafficking through the plant.

### Evidence of IR association with CP

As already stated, IR-carrying constructs are induced to move systemically and express inserted sequences following challenge inoculation with TYLCV. CP has been indicated (along with V2) as a factor involved in virus mobility. Immunocapture-PCR assays were performed with IR–PDS as the template (PDS is easily distinguished from CP). The PCR tubes were coated with anti-TYLCV–CP antibodies. Extracts of IR–PDS-treated, TYLCV-infected plants were incubated in the coated tubes, and unbound material was washed away. The coated tubes were subjected to DNase treatment and UV irradiation to remove any DNA bound to the outside of the trapped entities, and PCR was carried out with primers for the PDS gene.

**Fig. 9:**
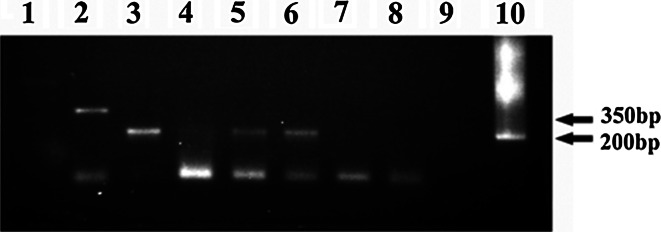
Immunocapture assays of plants treated with IR–PDS. Lane 1, PCR of the antibody-bound fraction from a TYLCV-infected, untreated plant; lane 2, PCR of the antibody-bound fraction from IR–PDS-treated plants with primers for TYLCV CP; lanes 3–6, same as in lane 2 but with primers for PDS amplification; lanes 7 and 8, negative controls—PCR with plant extracts in the absence of anti-TYLCV CP antibodies; lane 9, empty; lane 10, positive control—a PDS-carrying plasmid served as the template

Aside from the expected trapped TYLCV, the immunocaptured material also included PDS DNA (Fig. 9). Neither PDS nor TYLCV was amplified in the absence of antibodies, ruling out the possibility that amplification resulted from plant material not associated with CP. Thus, TYLCV infection appears to provide the capsid part for the IR–PDS association with CP. The association with *in-trans*-provided CP suggests that at least some of the roles of the IR are carried out by IR–CP complexes.

## Discussion

It was previously impossible to determine the functional phase of the TYLCV “life cycle” at which a certain event occurs. Using the IL-60 system [17], however, dsDNA replication and expression can be uncoupled from the normal “life cycle” of TYLCV [ssDNA→(dsDNA→dsDNA→dsDNA)_n_→ssDNA]. This enables studying events occurring in the middle phase (dsDNA and its various functions) and their separation from early-phase events (entry, uncoating, transport to the nucleus, etc.) and late-phase functions (replication of progeny ssDNA, virus assembly, transmission to other plants). Many pieces of data provided in this paper are restricted to the middle phase of the TYLCV “life cycle”. Employing the IL-60 system, we reveal new features of the noncoding (IR) component of the TYLCV genome and revise the role of some of the other TYLCV genes.

The small-size TYLCV exerts its activities mostly by recruiting host factors through protein-protein and protein-DNA interactions [25–31]. These interactions are sometimes regulated by interactions between the viral products themselves. In several cases, virus-host complexes increase due to induced expression of the host proteins participating in complex formation or of their regulatory elements [32–35]. Some of the roles of viral genes, discussed further on, involve complexes of viral gene products or viral sequences with host elements.

### Function of complementary-oriented TYLCV gene products

Only the IR and the sense-oriented genes *V1* and *V2* are required for replication, expression and movement. However, the complementary-oriented genes (*C1–C4*) are known to have a role in replication and expression as well. This seeming contradiction can be explained by separating the various roles along a time line. During the early events, basal levels of the viral genes are expressed. Some of the gene products interact among themselves or with host factors, producing complexes that will serve in a later phase when progeny viral particles are produced. As already noted, a viral gene product (CP) interacts with the IR (Fig. 9). The IR-CP complex may enable TYLCV infection to proceed to the next stage (i.e., late events).

Antisense-oriented viral genes have been reported to be associated with DNA replication [30 and citations therein]. However, as shown, none of the complementary-oriented TYLCV gene products are required for dsDNA replication. Nevertheless, C3, the product of ORF *C3* of TYLCV, has been characterized as a DNA-replication enhancer. These seemingly contradictory data might be explained by taking into account that these activities can occur at different phases of TYLCV replication. C3 affects the rolling-circle phase and replication of progeny viral ssDNA, probably by interaction with C1 and by self-oligomerization [30]. Despite being termed “DNA-replication enhancer”, it is not involved in, or required for, any aspect of dsDNA replication in the middle phase.

Complementary-oriented viral gene products (C1, C3, C4) are involved in recruiting host factors that restore cell-cycle programming in resting plant cells [25–31] and stimulate the expression or activation of proteins of the transduction pathway [31, 36]. C3 interacts with the host proliferating cell nuclear antigen (PCNA), which is involved in normal cell-cycle processes and might therefore affect dsDNA replication at a later phase. Virus-induced reprogramming of the expression of host genes and the resultant interactions between viral and host-cell components might be required for the acceleration of dsDNA replication in the late phase, when many progeny ssDNA are produced from dsDNA and the production of template dsDNA is accelerated as well.

C2 has been identified as a transcriptional activator of late viral gene expression and a silencing suppressor. C2 analogs in other geminiviruses have also been reported to be transcriptional activators [37–39]. However, with C2 attached (Fig. 1) or in the absence of C2, the construct IR–V2–V1 drives expression to similar levels (data not shown). Hence, C2 is not required for activation of the basal transcription of sense-oriented genes. These data may not be contradictory: the presently studied middle-phase activities of TYLCV require only factors for dsDNA replication, expression and movement. In the late phase, on the other hand, many CP molecules are required for progeny virus encapsidation, and the rate of CP production must be considerably increased. Thus, C2 produced in the middle phase has no role in middle-phase activities but is required for the late phase of TYLCV infection; with its binding capacity to ssDNA [38], C2 may be regulated by the accumulated ssDNA and/or by the IR–CP complex, thus elevating transcription rates to accommodate the increased need for CP in the late phase.

### Role of IR in DNA longevity and replication

The IR was shown to be a stabilizing element for dsDNA. One possible reason might be that it attracts DNA-binding host proteins to construct a protective shield. Indeed, geminiviral dsDNA has been reported to associate with histones to form minichromosomes [40, 41], and the IR may mediate this process. Another possibility is that IR–X (X being any DNA) stability is gained by its autonomous replication. Our findings support the latter notion that dsDNA “stabilization” (increased longevity) by IR is due to perpetual replication.

We demonstrated that IR is the only viral component required for dsDNA replication. Since replication does not require any viral gene product, it must be entirely dependent on the recruitment of host factors. Presumably, the IR attracts the host factors, initiating the replication of IR-carrying DNA, which then becomes replication-competent.

### Expression

None of the geminivirus (including TYLCV) genes code for RNA polymerases. Hence, viral transcription depends entirely on host polymerases and transcription factors that recognize the promoters located in the IR. Here, we demonstrate that the minimal requirement for expression (transcription and translation) is the two IR-directed sense-oriented TYLCV genes *V1* and *V2*. Foreign genes fused to IR–*V2–V1*, such as with p1470–GFP (Fig. 1), are also expressed. Foreign genes directed by the IR would also be expressed *in trans*, i.e., gene X would replicate as an IR–X construct but would be expressed only if supplemented *in trans* by p1470. None of the complementary-sense TYLCV genes seem to be essential for expression, replication, or movement. Nevertheless, they might affect the levels of replication and expression due to interactions with host factors or direct association with IR promoters.

The ORFs V1 and V2 were found to be necessary for both expression and movement. These two aspects may be linked together. It is possible that the initial expression took place in the primary cells and the entities spreading to distant cells were the transcripts. In such a case, movement would be an automatic consequence of expression.

### Movement

It is well established that the karyophilic CP facilitates TYLCV ssDNA import into the nucleus [11, 16, 42, 43]. Its spread, however, depends on export of the viral DNA to the cytosol, movement to the cell periphery and traversing to neighboring cells. CP has indeed been reported to carry nuclear export signals as well, and to mediate the nuclear exit of viral DNAs [10, 42, 43], which move within the plant in both their ss and ds forms [9]. Transport from the nucleus to the cytoplasm and cell-to-cell trafficking through plasmodesmata have been suggested to be mediated by various CP–protein complexes [16, 44]. The level of expression of CP from IR–*V1*–*V2* is sufficient for viral DNA trafficking and possibly, when combined with host factors and/or viral DNA sequences, the initiation of late viral functions as well. The products of *C2* and *C4*, as well as some host proteins, have also been reported to be involved in virus trafficking [45, 46]. However, our results show that the sense-oriented viral genes (*V1*, *V2*) are sufficient for movement of viral dsDNA within the plant. It has been suggested that both ssDNA and dsDNA are shuttled within the infected plant. C2 and C4 may play a role in ssDNA movement and be redundant for dsDNA shuttling.

As discussed above, the moving entities could be the transcripts made following the enabling of expression. Thus, the initiation of expression in one cell allows the spread of the expressing entity to other cells. If this is the case, the “expression” and “movement” are cause and consequence.

### Other features of the IR

As shown in this report, the IR, even when fused to a sequence foreign to the TYLCV system, binds CP. All IR-X constructs in this paper are dsDNA. Hence, the conclusion is that the IR in its dsDNA form is associated with CP. dsDNA is not encapsidated, making it unlikely that such an interaction is required for virion assembly. As reported here and elsewhere, CP is probably involved in dsDNA movement. It is therefore likely that CP binds to the IR in its dsDNA form. We thus propose that CP has a regulatory role in one or more IR functions: for instance, enhancement of IR promoter activity, better driving of *V2–V1* expression following CP binding, and the formation of a positive loop in which the CP produced at an early stage enhances its own production when more CP is needed at a later stage of the viral “life cycle”. A similar role was assigned to the gene product of *C2*, which, in complex with host proteins, exerts late promoter activity, probably, as indicated in this report, by interacting with IR–CP.

### From a satellite to a replication-competent plant plasmid and virus

Many naturally occurring geminivirus-associated satellites have been isolated and characterized (e.g., see Briddon and Stanley [47]). Most of them are about half the size of the helper virus genome, except for the very short (682 bp) satellite described by Dry et al. [48]. All hitherto-described geminivirus-related satellites carry motifs that are also in the helper virus IR. They are encapsidated and insect-transmissible. Here, we further developed IL-60–BS [17] into a shorter construct (p1470), carrying only the IR and two viral genes, that replicates, expresses and moves independently of all other viral components. P1470 can therefore be considered a plant plasmid. For expression, p1470 relies on a helper virus, or parts thereof, and hence can be considered a satellite.

It appears that the two parts of the TYLCV genome assume different sets of functions. The sense-oriented genes are involved in all matters concerning the intraplant dsDNA phase of infection as expected of a plasmid, i.e., stability, replication, expression and movement. Thus, independent of the complementary-oriented part of the genome, the sense-oriented segment can develop into an autonomous entity capable of moving within the plant. However, it cannot produce ssDNA progeny or assemble into virions. Hence, it is unable to be naturally transmitted from one plant to another. The complementary-oriented genes are required for conversion of the dsDNA to its progeny ssDNA (by rolling-circle replication) and also have roles in recruiting plant factors that aid in replication and expression. The TYLCV progeny ssDNA, however, can be encapsidated, and the resultant viral particles are transmissible from one plant to another by insects. Hence, the complementary-oriented part of the TYLCV genome enables conversion of a plasmid to a virus.

## Electronic supplementary material

Below is the link to the electronic supplementary material.

Supplementary material 1 (DOCX 13 kb)

## References

[1] Stanley J (1985). The molecular biology of geminiviruses. Adv Virus Res.

[2] Stanley J, Bisaro DM, Briddon RW, Brown JK, Faucuet CM, Harrison BD, Faucuet CM, Maniloff J, Desselberger U, Ball LA (2005). Family geminiviridae. Virus taxonomy. English report of the International Committee on Taxonomy of Viruses.

[3] Argüello-Astorga GR, Guevara-González RG, Herrera-Estrella LR, Rivera-Bustamante RF (1994). Geminivirus replication origins have a group-specific organization of iterative elements: a model for replication. Virology.

[4] Navot N, Pichersky E, Zeidan M, Zamir D, Czosnek H (1991). Tomato yellow leaf curl virus—a whitefly-transmitted geminivirus with a single genomic component. Virology.

[5] Jeske H (2009). Geminiviruses. Curr Top Microbiol Immunol.

[6] Hanley-Bowdoin L, Settlage SB, Orozco BM, Nagar S, Robertson D (1999). Geminiviruses: models for plant DNA replication, transcription, and cell cycle regulation. Crit Rev Plant Sci.

[7] Gutierrez C (1999). Geminivirus DNA replication. Cell Mol Life Sci.

[8] Singh DK, Malik PS, Chaudhury NR, Mukherjee SK (2008). MVMIV replication initiator protein (Rep): roles at the initiation and elongation steps of MVMIV DNA replication. Virology.

[9] Rojas MR, Noueiry AO, Lucas WJ, Gilbertson RL (1998). Bean dwarf mosaic geminivirus movement proteins recognize DNA in a form- and size-specific manner. Cell.

[10] Rojas MR, Jiang H, Salati R, Xoconostle-Cázares B, Sudarshana MR, Lucas WJ (2001). Functional analysis of proteins involved in movement of the monopartite begomovirus, Tomato yellow leaf curl virus. Virology.

[11] Gafni Y, Epel BL (2002). The role of host and viral proteins in intra- and inter-cellular trafficking of geminiviruses. Physiol Mol Plant Pathol.

[12] Jeffrey JL, Pooma W, Petty ITD (1996). Genetic requirements for local and systemic movement of Tomato golden mosaic virus in infected plants. Virology.

[13] Liu H, Boulton MI, Thomas CL, Prior DAM, Oparka KJ, Davis JW (1999). Maize streak virus coat protein is karyophyllic and facilitates nuclear transport of viral DNA. Mol Plant Microbe Interact.

[14] Noueiry AO, Lucas WJ, Gilbertson RL (1994). Two proteins of a plant DNA virus coordinate nuclear and plasmodesmal transport. Cell.

[15] Pascal E, Sanderfoot AA, Ward BM, Medville R, Turgeon R, Lazarowitz SG (1994). The geminivirus BR1 movement protein binds single-stranded DNA and localizes to the cell nucleus. Plant Cell.

[16] Sharma P, Ikegami M (2009). Characterization of signals that dictate nuclear/nucleolar and cytoplasmic shuttling of the capsid protein of Tomato leaf curl Java virus associated with DNAβ satellite. Virus Res.

[17] Peretz Y, Mozes-Koch R, Akad F, Tanne E, Czosnek H, Sela I (2007). A universal expression/silencing vector in plants. Plant Physiol.

[18] Mozes-Koch R, Gover O, Tanne E, Peretz Y, Maori E, Chernin L, Sela I (2012). Expression of an entire bacterial operon in plants. Plant Physiol.

[19] Sambrook J, Russell RD (2001). Molecular cloning.

[20] Jefferson RA, Kavanagh TA, Bevan MW (1987). GUS fusions: beta-glucuronidase (gus) as a sensitive and versatile gene fusion marker in plants. EMBO J.

[21] Xi JJ, Xu W, Shanyu L, Xin Z, Lin Y, Jia F, Dongyun H (2006). Polyethylene glycol fractionation improved detection of low-abundant proteins by two-dimensional electrophoresis analysis of plant proteome. Phytochemistry.

[22] Nagata T, Nemoto Y, Hasezawa S (1992) Tobacco BY-2 cell line as the “HeLa” cell in the cell biology of higher plants. Kwang WJ, Martin F (eds) International review of cytology. Academic Press, pp 1–30

[23] Wetzel T, Candresse T, Macquaire G, Ravelonandro M, Dunez J (1992). A highly sensitive immunocapture polymerase chain-reaction method for plum pox potyvirus detection. J Virol Meth.

[24] Poogin MM (2013). How can plant DNA viruses evade siRNA-directed DNA methylation and silencing. Int J Mol Sci.

[25] Gutierrez C (2000). DNA replication and cell cycle in plants: learning from geminiviruses. EMBO J.

[26] Gutierrez C, Ramirez-Parra E, Castellano MM, Sanz-Burgos AP, Luque A, Missich R (2004). Geminivirus DNA replication and cell cycle interactions. Vet Microbiol.

[27] Hanley-Bowdoin L, Settlage SB, Robertson D (2004). Reprogramming plant gene expression: a prerequisite to geminivirus DNA replication. Mol Plant Pathol.

[28] Lai JB, Chen H, Teng KL, Zhao QZ, Zhang ZH, Li Y (2009). RKP, a RING finger E3 ligase induced by BSCTV C4 protein, affects geminivirus infection by regulation of the plant cell cycle. Plant J.

[29] Settlage SB, Miller AB, Gruissen W, Hanley-Bowdoin L (2001). Dual interaction of geminivirus replication accessory factor with a viral replication protein and a plant cell cycle regulator. Virology.

[30] Settlage SB, See RG, Hanley-Bowdoin L (2005). Geminivirus C3 protein: replication enhancement and protein interactions. J Virol.

[31] Shen W, Reyes MI, Hanley-Bowdoin L (2009). Arabidopsis protein kinases GRIK1 and GRIK2 specifically activate SnRK1 by phosphorylating its activation loop. Plant Physiol.

[32] Baliji S, Lacatus G, Sunter G (2010). The interaction between geminivirus pathogenicity proteins and adenosine kinase leads to increased expression of primary cytokinin-responsive genes. Virology.

[33] Caracuel Z, Lozano-Duran R, Huguet S, Arroyo-Mateos M, Rodriguez-Negrete EA, Bejarano ER (2012). C2 from Beet curly top virus promotes a cell environment suitable for efficient replication of geminiviruses, providing a novel mechanism of viral synergism. New Pathol.

[34] Lozano-Duran R, Bejarano R (2011). Geminivirus C2 protein might be the key player for geminiviral co-option of SCF-mediated ubiquitination. Plant Signal Behav.

[35] Trinks D, Rajeswaran R, Shivaprasad PV, Akbergenov R, Oakeley EJ, Velutambi K (2005). Suppression of RNA silencing by a geminivirus nuclear protein, AC2, correlates with transactivation of host genes. J Virol.

[36] Ascencio-Ibáñez JT, Sozzani R, Lee T-J, Chu T-M, Wolfinger RD, Cella R, Hanley-Bowdoin L (2008). Global analysis of Arabidopsis gene expression uncovers a complex array of changes impacting pathogen response and cell cycle during geminivirus infection. Plant Physiol.

[37] Collin S, Fernandez Lobato M, Gooding PS, Mullineaux PM, Fenoll C (1996). The two nonstructural proteins from wheat dwarf virus involved in viral gene expression and replication are retinoblastoma-binding proteins. Virology.

[38] Hartitz MD, Sunter G, Bisaro DM (1999). The Tomato golden mosaic virus transactivator (TrAP) is a single-stranded DNA and zinc-binding phosphoprotein with an acidic activation domain. Virology.

[39] Sunter G, Bisaro DM (1997). Regulation of a geminivirus coat protein promoter by AL2 protein (TrAP). Evidence for activation and derepression mechanisms. Virology.

[40] Pilartz M, Jeske H (1992). Abutilon mosaic geminivirus double-stranded DNA is packed into minichromosomes. Virology.

[41] Pilartz M, Jeske H (2003). Mapping of Abutilon mosaic geminivirus minichromosomes. J Virol.

[42] Kunik T, Palanichelvam K, Czosnek H, Citovsky V, Gafni Y (1998). Nuclear import of the capsid protein of tomato yellow leaf curl virus (TYLCV) in plant and insect cells. Plant J.

[43] Liu H, Boulton MI, Thomas CL, Prior DAM, Oparka KJ, Davis JW (1999). Maize streak virus coat protein is karyophyllic and facilitates nuclear transport of viral DNA. Mol Plant Microbe Interact.

[44] Hehnle S, Wege C, Jeske H (2004). Interaction of DNA with movement proteins of geminiviruses revised. J Virol.

[45] Jupin I, De Kouchkovsky F, Jouanneau F, Gronenborn B (1994). Movement of Tomato yellow leaf curl geminivirus (TYLCV): involvement of the protein encoded by ORF C4. Virology.

[46] Teng KL, Chen H, Lai J, Zhang Z, Fang Y, Xia R (2010). Involvment of C4 protein of Beet severe curly top virus (familiy Geminiviridae) in virus movement. PLoS One.

[47] Briddon RW, Stanley J (2006). Subviral agents associated with plant single-stranded DNA viruses. Virology.

[48] Dry IB, Krake LR, Rigden JE, Rezaian MA (1997). A novel subviral agent associated with a geminivirus: the first report of a DNA satellite. Proc Natl Acad Sci USA.

