# Supplementary material for: Only minimal regions of tomato yellow leaf curl virus (TYLCV) are required for replication, expression and movement
Source: Arch Virol. 2014 Apr 10;159(9):2263–74. doi: 10.1007/s00705-014-2066-7 (PMC4147252; doi:10.1007/s00705-014-2066-7)
Supplement: Supplementary file 1 — Supplementary material 1 (DOCX 13 kb) [file 705_2014_2066_MOESM1_ESM.docx]

| Primer name | Sequence 5’→3’ | Target gene position |
| --- | --- | --- |
| 1465 | CTGATAGCGCGTGACAAAA | *uidA* GenBank acc. # M14641, bp: 2648–2666 |
| 1466 | CGGTTCGTTGGCAATACTC | *uidA* GenBank acc. # M14641, bp: 2707–2689 |
| 1546 | TGGAGCGATTTGTCTGGTTA | *Nicotiana tabacum*18S GenBank acc. # HQ384692.1,  bp: 1277–1296 |
| 1547 | AAGGGATACCTCCGCATAGC | *Nicotiana tabacum*18S GenBank acc. # HQ384692.1,  bp: 1304–1322 *Nicotianatabacum* 18S Gene bank acc: HQ384692.1, bp: 1352-1333 |
| 734 | ATTGATCAGCGTTGGTGGGA | Forward primer for sequencing of GUS. Located 70 bp downstream to the first ATG. Bases 1553-1573 of **gus #M14641***uida*Gene bank acc: #M14641, bp: 1553-1573 |
| 735 | TGCGGTCGCGAGTGAAGATC | Reverse primer, located 1712bp downstream to the first ATG. Bases 3198-3178 of **gus #M1464**  (Opposite direction to # 261 primer) ***Tm 71^0^C*** *UidA Gene bank acc:* #M14641, bp: 3198-3178 |
| 331 | TATAAACTACAGAAAAGC | Reverse flanking GUS intron |
| 1268 | GGAGAGGGTGAAGGTGATGC | GFP GenBank acc. # U87974, bp 1–20 |
| 1269 | GTCACGCTTTTCGTTGGGAT | GFP GenBank acc. # U87974, bp 777–797 |
| 100 | CTATCCTTCGCAAGACCCTCC | 35S promoter GenBank acc. # JF809616.1,  bp: 7374–7392 |
| 170 | TGAATGCGCGAAACAAG | 35S promoter GenBank acc. # JF809616.1,  bp: 7553–7535 |
| 966 | ATTGGGCTGTTTCCATAGGGC | TYLCV*-CP* GenBank acc. # JX131286.1,  bp: 759–739 |
| 967 | GAAGGCTGAACTTCGACAG | TYLCV*-CP* GenBank acc. # JX131286.1,  bp: 360–378 |
| 1117 | CTAAATACTCTTAAGAAATGACCAGTCTGAGGCTG | TYLCV*-C2* GenBank acc. # JX131286.1,  bp: 1223–1257 |
| 1118 | AAGAATGCAACCTTCGTCACCCTCTAC | TYLCV*-C2* GenBank acc. # JX131286.1,  bp*:* 1634–1608 |
| Roche probe #9 | CTGATAGCGCGTGACAAAAACCACCCAAGCGTGGTGATGTGGAGTATTGCCAACGAACCF | *uidA* GenBank acc. # M14641, bp: 2658–2718 |
| Roche probe #111 | TGGAGCGATTTGTCTGGTTAATTCCGTTAACGAACGAGACCTCAGCCTGCTAACTAGCTATGCGGAGGTATCCCTT | *Nicotiana tabacum*18S  GenBank acc. # HQ384692.1, bp: 1277–1358 |
